# Editorial: Long non-coding RNAs in lung cancer

**DOI:** 10.3389/fonc.2022.1100061

**Published:** 2022-12-12

**Authors:** Umberto Malapelle, Giulia Veronesi

**Affiliations:** ^1^ Department of Public Health, University of Naples Federico II, Naples, Italy; ^2^ Department of Thoracic Surgery, Istituto di Ricerca e Cura a Carattere Scientifico (IRCCS) San Raffaele Scientific Institute, Milan, Italy; ^3^ School of Medicine, Vita-Salute San Raffaele University, Milan, Italy

**Keywords:** lncRNA, lung cancer, molecular pathology, molecular oncology, liquid biopsy

Lung cancer remains the leading cause of cancer-related deaths worldwide (1). Currently, lung cancer patients show poor clinical outcomes primarily due to the challenges of early detection, the high risk of metastasis, and the development of resistance to multiple therapies. Novel molecular markers are currently under investigation not only for predictive but also for prognostic purposes. Among these, an interesting field of investigation is long non-coding RNAs (lncRNAs). LncRNAs are RNA molecules composed of more than 200 nucleotides that, despite not being involved in protein generation, play a pivotal role in gene expression regulation (2). This Frontiers in Oncology Research Topic addresses some major concerns related to the prognostic role of lncRNAs in lung cancer patients.

The role of lncRNA GCC2-AS1 in primary malignant tumors is still debated. However, Yu et al. have highlighted that GCC2-AS1 expression was significantly up-regulated in lung adenocarcinoma with respect to normal tissues. Interestingly, the authors demonstrated that the depletion of GCC2-AS1 inhibited the proliferation and invasion of lung adenocarcinoma cells *in vitro*. This provides strong evidence that an elevated level of GCC2-AS1 in these patients might indicate a poor prognosis.

In another experiment, Khadirnaikar et al. were able to identify a novel subtype of lung adenocarcinoma, namely, undifferentiated lung adenocarcinoma, by adopting a set of embryonic stem cell lncRNAs. This sub-class features high stem cell-like characteristics and poor clinical outcomes and may be referred for immunotherapy as a first-line treatment.

Another crucial role played by lncRNAs is the regulation of ferroptosis, which is an iron-dependent cell death mechanism that is important in the survival of tumor cells, as reported by Lu et al. In addition, lncRNAs may be involved in maintaining genomic stability. By combining the lncRNA expression profiles associated with somatic mutations and the corresponding clinical characteristics of lung adenocarcinoma, Yang et al. were able to generate a lncRNA signature related to genomic instability.

Interestingly, it has been demonstrated that lncRNAs can interact with micro RNAs (miRNAs), thereby affecting and regulating the expression of target genes and influencing the outcome in lung adenocarcinoma patients (Wu et al.; Fan et al.).

Another frontier in this field is the possibility of extracting lncRNAs from liquid biopsy samples. In this scenario, extracellular vesicles and exosomes may play a crucial role in obtaining high quality lncRNAs as biomarkers for predictive and prognostic purposes in lung adenocarcinoma patients (Fan et al.).

This Research Topic highlights the role of lncRNAs in lung cancer and in particular in lung adenocarcinoma patients. Further research is warranted to improve the clinical outcome of these patients.

## Author contributions

All authors listed have made a substantial, direct, and intellectual contribution to the work and approved it for publication.

## References

[1] Siegel RL Miller KD Fuchs HE Jemal A. Cancer statistics, 2022. CA Cancer J Clin (2022) 72:7–33. doi: 10.3322/caac.21708 35020204

[2] Statello L Guo CJ Chen LL Huarte M. Gene regulation by long non-coding RNAs and its biological functions. Nat Rev Mol Cell Biol (2021) 22:96–118. doi: 10.1038/s41580-020-00315-9 33353982 PMC7754182

